# Antischistosomal Potential of Animal-Derived Natural Products and Compounds

**DOI:** 10.3390/microorganisms13020397

**Published:** 2025-02-11

**Authors:** Agatha Fischer-Carvalho, Tereza Cristina Taveira-Barbosa, Sergio Verjovski-Almeida, Simone Haeberlein, Murilo Sena Amaral

**Affiliations:** 1 Laboratório de Ciclo Celular, Instituto Butantan, São Paulo 05503-900, SP, Brazil; a.carvalho.proppg@proppg.butantan.gov.br (A.F.-C.);; 2 Instituto de Química, Universidade de São Paulo, São Paulo 05508-900, SP, Brazil; 3 Biomedizinisches Forschungszentrum Seltersberg, Institute of Parasitology, Justus Liebig University Giessen, Schubertstr. 81, 35392 Giessen, Germany

**Keywords:** schistosomiasis, animal natural products, antiparasitic activity

## Abstract

Schistosomiasis is a neglected tropical disease that affects over 240 million people worldwide. Currently, praziquantel is the only drug recommended by the World Health Organization for treatment. However, cases of drug resistance have been reported, which indicates an urgent need for new therapeutics. In this context, natural compounds represent valuable sources of pharmacological substances. Plant-derived natural products have been greatly explored for their potential antischistosomal activity, while animal-derived compounds have received little attention. Recent advances in the biotechnology field allow the wide exploration of animal-derived compounds in drug discovery, which may represent a cost-effective option to find bioactive molecules also against *Schistosoma mansoni* and other parasites. This review highlights the research into animal-derived products and compounds that have already been tested against schistosomes. Phenotypic effects on schistosomes have been observed upon incubation with some of these substances, which may, therefore, represent possible candidates to be used in the development of new drugs. Overall, these studies advance the discovery of antischistosomal compounds by exploring a yet understudied natural resource. The present review also discusses the challenges of testing animal-derived products and provides examples of the experimental in vitro testing of different selected animal natural products against *S. mansoni*.

## 1. Introduction

Schistosomiasis is a neglected tropical disease caused by parasitic trematodes of the genus *Schistosoma*, with three species (*S. mansoni*, *S. haematobium*, and *S. japonicum*), presenting greater medical relevance. It affects approximately 240 million people over 78 countries; in 51 of them, the disease is considered endemic from moderate- to high-transmission and preventive chemotherapy is applied [1]. In addition, it is responsible for over 280,000 human deaths per annum in sub-Saharan Africa alone [2,3]. Predominating in tropical and subtropical areas, it is estimated that 90% of infections occur in sub-Saharan Africa, especially in regions with a lack of sanitation [4,5].

**Figure 1:**
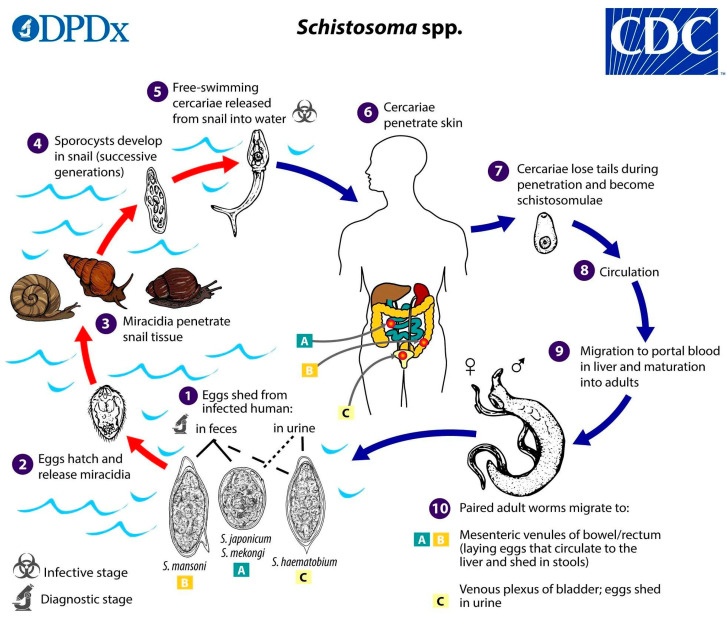
Life cycle of *Schistosoma* spp. Image from the Centers for Disease Control and Prevention (CDC). Available at https://www.cdc.gov/dpdx/schistosomiasis/index.html, accessed on 29 December 2024.

The different *Schistosoma* species are dioecious blood-flukes that have complex heteroxenic life cycles (Figure 1), involving invertebrate hosts (freshwater snails) and vertebrate hosts, usually mammals [6]. Human infection occurs after the penetration of cercariae, the larval stage shed by infected snails, through the skin after contact with contaminated fresh water. Along this process, cercariae lose their tails, becoming the immature forms of the parasite (schistosomula) that reach the bloodstream, develop into adult male and female worms, and mate. After 5–7 weeks, *S. mansoni* and *S. japonicum* adult female worms start producing eggs, which are released into the mesenteric veins of the intestine.

The tissue deposition of eggs is the main factor responsible for the disease pathology, which includes symptoms such as abdominal pain, diarrhea, granulomatous disease, hepatosplenomegaly, and other disabling systemic morbidities, including anemia and impaired childhood development [6,7]. A fraction of the eggs can be expelled in the feces, reach the environment, release ciliated miracidia that will infect the snail intermediate host, and, after asexual reproduction, shed cercariae into the water, continuing the cycle [3].

Besides the disease pathology, schistosomiasis often occurs parallel to other diseases, including malaria, tuberculosis, and HIV infections [3,8,9]. Coinfections can increase the severity of the symptoms and morbidity. This not only has a high impact on public health, and high costs for the public health system, but also impacts the economic development due to the disease chronicity and disability [1]. Currently, praziquantel (PZQ) is the only drug recommended for schistosomiasis treatment, and it has been used in mass drug administration programs [1,10], which represents a rising concern in the establishment of drug-resistant parasites. In fact, cases of parasites tolerant to PZQ in the field have already been described [11,12,13,14], and PZQ-resistant strains have been generated in the laboratory [15,16,17,18]. In addition, although PZQ represents a low-cost drug with good efficacy and tolerability, it has limited efficacy over schistosomula.

Having a single drug as chemotherapy for schistosomiasis represents a great concern and demonstrates the urgent need for new therapeutics. In fact, the WHO has recently concluded that praziquantel mass drug administration as a single intervention may not be adequate for eliminating schistosomiasis globally [19]. Therefore, having alternative therapeutic compounds will both increase the possibility of disease control and reduce the chances of the development of resistant parasite strains. In this direction, natural compounds have been explored as sources of new biologically active molecules [20], and a few reviews have already discussed this research field in the context of antischistosomal drug discovery [21,22], largely focusing on plant-derived compounds with an anthelmintic capacity. On the other hand, animal-derived compounds have received little attention against schistosomes, even though they have been the focus of drug discovery for several other therapeutic applications [23,24]. This review will focus on several animal-derived products and compounds that have already been tested against schistosomes, showing good potential as sources of new schistosomicide molecules. Furthermore, we show examples from our own work focusing on the in vitro testing of selected natural products from four different animals against *S. mansoni*.

## 2. Animal Natural Products as Sources of New Antischistosomal Compounds

Natural products (NPs), especially the ones derived from plants, have been proposed as possible sources of new drugs against helminths, including *Schistosoma* [21,22]. This occurs in part because NPs are considered great sources of bioactive molecules. Over the past four decades, approximately 42% of all approved drugs have either been classified as NPs or derived from these sources [25]. Ivermectin, a semi-synthetic drug derived from lactones produced by *Streptomyces avermitilis*, is used against onchocerciasis, strongyloidiasis, and other geohelminthiasis [26], and represents a prominent example of an anthelmintic drug derived from NPs.

Plant-derived NPs have been greatly explored in research for their potential antischistosomal activity, with significant success, although no drugs have been approved until now [21,22]. One of the NPs is artemisinin, already known for its antimalarial activity, which has been also proposed as a potential treatment and prophylaxis against schistosomiasis and other helminth infections [27,28,29]. On the other hand, animal-derived NPs against schistosomes still represent an underestimated topic in the literature, although they may constitute valuable resources for therapeutic applications, showing antibacterial and antitumor potentials [30,31].

The animal kingdom encompasses a vast biodiversity with numerous species still undiscovered, and it remains largely underutilized for bioactive natural product exploration. Different substances have been produced by them as natural weapons for self-defense, and might be used as tools for drug development. As an example, antimicrobial peptides (AMPs), key components of the innate immune system, can rapidly recognize and eliminate pathogens [32]. Despite their widespread presence across various organisms as part of their defense strategies, only one AMP has been formally reported for its efficacy against schistosomes [33].

Rich in polysaccharides and peptides, other animal-derived substances, such as natural secretions, have been explored for their biomedical effects, including antibacterial applications [34]. Bee extracts, such as propolis and honey, have been used for centuries for different therapeutic purposes [35]. In contrast, bioactive compounds derived from arthropods and marine organisms seem to represent a relatively new frontier, yet remain largely underexplored in the context of parasitic diseases, particularly schistosomiasis [36,37].

The most explored category of animal-derived compounds against parasites is venoms. Some snake, scorpion, and arthropod venoms [38,39] caused *Leishmania* ultrastructural alterations and growth inhibition [40,41,42]. Other studies performed the purification of proteins [43] and investigation of immunostimulatory mechanisms in response to *Leishmania* parasite infection [44]. Meanwhile, the antischistosomal activity of venoms is still poorly understood, and only a few works have evaluated the effect of venoms or their components on *S. mansoni* [33,37,45,46,47,48]. Venoms are composed of a large variety of enzymatic and non-enzymatic pharmacologically active substances, such as proteins, polypeptides, carbohydrates, lipids, metal ions, and other unidentified compounds [24]. These complex mixtures represent a substantial source of new therapeutics [49]. However, only a few compounds present in venoms have been shown to be responsible for its toxicity as discussed below, which emphasizes the need of new studies for the further optimization of their effectiveness against parasites.

## 3. Venoms as Sources of Therapeutic Compounds

Venoms represent a wealthy source of pharmacologically active biomolecules, which can be found in numerous organisms, both invertebrates and vertebrates. Their diversity and potency are a reflection of thousands of years of refinement led by the evolutionary process, representing “preoptimized” molecules [50]. One of the pioneer and most successful examples of the use of an animal venom in drug discovery was the development of the antihypertensive captopril, a synthetic molecule built as a mimic of bradykinin-potentiating peptides from *Bothrops jararaca* [51].

The first and still ongoing investigations on venoms intend to determine their composition and toxic mechanisms as a strategy to develop a serum against snakebites [24]. Since then, many venoms have been extensively described, and represent already valuable therapeutic tools for life saving [24,52,53]. In fact, molecules found in animal venoms are known for affecting hemostasis and thrombosis [54,55,56], for having cytotoxic activity [57], and for damaging the extracellular matrix [58], among other effects. However, cellular toxicity and nonspecific effects were a major hindrance to exploring venoms as sources of new anthelmintic compounds. Nowadays, significant technical improvements enabled the screening and purification of venom components, making them a suitable source for therapeutic prospection. Against *S. mansoni*, a few crude venoms from different organisms have been tested, such as from snakes and insects [37,46,47,48]. Nevertheless, only a limited number of compounds with antischistosomal activity have been identified in these venoms, underscoring the urgent need for further research to discover their components and refine their efficacy.

## 4. Animal-Derived Products and Their Antischistosomal Potential

**Figure 2:**
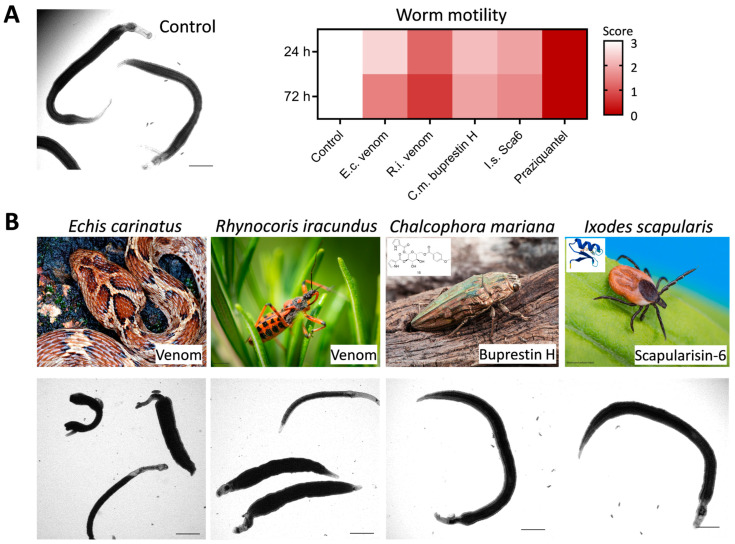
Antischistosomal activity of animal-derived natural products. (**A**) Adult *S. mansoni* couples were treated for 72 h with crude products or purified compounds and motility was assessed by microscopy (Score 3, normal motility, down to Score 0, dead) at 24 h and 72 h. Four different animal-derived natural products were assayed: venom from saw-scaled viper *Echis carinatus* (E.c.) at 20 µg/mL; venom from the assassin bug *Rhynocoris iracundus* (R.i.) at 50 µg/mL; buprestin H, an acyl glucose derivative, from the European jewel beetle *Chalcophora mariana* (C.m.) at 40 µM; and the defensin scapularisin-6 from the black-legged tick *Ixodes scapularis* (I.s.) at 50 µM. As a positive control, 2 µM praziquantel was used. Means of two independent replicates. (**B**) Source organisms and representative images of adult worms treated for 72 h. Scale bar: 500 µm.

In this section, we will describe ten published studies that explored the antischistosomal activity of animal-derived products (summarized in Table 1), taking into consideration their major effects. Furthermore, we provide example data from our own in vitro screens of NPs derived from four different taxa of animals (Figure 2A). The source organisms and representative images of treated adult *S. mansoni* worms after 72 h of incubation with the specified animal natural products are shown in Figure 2B.

### 4.1. Arthropod-Derived Products

#### 4.1.1. Scorpions

The first report of the use of animal-derived products against *S. mansoni* was published in 1980 by El-Asmar and collaborators [37]. In this work, the authors explored the impact of different scorpion venoms on the viability of *S. mansoni* cercariae. Previous studies had already identified substances in the venom that were toxic to insects and crustaceans and were shown to be different from mammalian toxins [59,60].

One drop of each of the venoms from the scorpions *Leiurus quinquestriatus*, *Buthus minax*, *Buthus occitanus*, and *Androctonus amoreuxi* was applied over approximately 20 cercariae in a petri dish. After 24 h, the cercariae demonstrated spastic behavior with the formation of granular precipitates [37]. Furthermore, the cercariae were ensheathed in a transparent envelope, which hardened gradually and restricted cercarial elasticity, leading to the release of the pericercarial envelope from the cercarial body [37]. The minimum amount of the crude venom to cause pericercarial envelope release was 10 µg per slide, while some fractions achieved a minimum concentration of 8 µg per slide. While the literature pointed to the presence of anticholinesterase activities in the venom of the scorpion *B. minax*, no anticholinesterase or proteolytic activity was detected in the active fractions of the tested *L. quinquestriatus* venom [37].

**Table 1:** Experimental details summary of published articles describing the effects of animal natural products on *Schistosoma mansoni*.

Organism	Natural Product	Reference	Year	Assay Type	Parasite Stage	Concentration	Duration of Incubation	Phenotype
Several species of scorpions	Venom solution or fraction	El-Asmar et al. [37]	1980	in vitro	Cercariae	Several concentrations	24 h	Spastic behavior, precipitate formation, elasticity restriction, and release of the pericercarial envelope.
Snake (*Bothrops moojeni*)	MjTX-II	Stábeli et al. [45]	2006	in vivo	Infected mice	50 and 100 μg	2 doses (0 and 20th day)	Reduction in the egg/gram, couple dissociation, inhibition on oviposition, and death of some parasites.
Frog (*Phyllomedusa*)	Dermaseptin 01	de Moraes et al. [33]	2011	in vitro	Adult worms	1–200 μg/ml	24–120 h	Increase in mortality, decrease in egg posture and motor activity, and tegument alterations.
79 marine organisms	Several substances	Melek et al. [61]	2012	in vitro	Adult worms	Several concentrations	4 days	Death of parasites.
Sea cucumbers	Holothurin extracts	Mona et al. [36]	2012	in vivo and in vitro	Adult worms	5.4, 10 and 62.2 mg/kg	24 h	Decrease in worm burden and liver egg count, tegument alterations, and death of parasites.
Bee (*Apis* *mellifera*)	Crude and dissolved venom; Propolis	Mohamed et al. [46]	2016	in vivo	Infected mice	1 bee sting, injected venom (0.1 mg/kg) or propolis doses (200 mg/kg)	Weekly for 2 weeks (venom) or 3 consecutive days (propolis)	Decrease in the number of worms, in ova count per gram in the liver, and in RNA percentage of exerted worms.
Snake (*Cerastes cerastes*)	Lyophilized venom	Hassan et al. [47]	2016	in vitro	Adult worms	10–50 μg/ml	24–72 h	Increase in mortality, destruction of the oral sucker, and alterations on tegument.
Ladybird (*Harmonia axyridis*)	Harmonine	Kellershohn et al. [62]	2019	in vitro	Adult worms	2.5–50 μM	24–72 h	Separation of couples, mortality increase, reduction in egg production, bubbles on the tegument, and inhibition of AChE enzymatic activity.
Assassin bug (*Rhynocoris iracundus*)	Crude venom	Tonk et al. [48]	2020	in vitro	Adult worms	10–50 μg/ ml	24–72 h	Detachment, decrease in mobility and egg production, depletion of proliferating stem cells, and intracellular degradation on the spermatogonia.
Jewel beetle (*Chalcophora mariana*)	Buprestin H	Gallinger et al. [63]	2022	in silico and in vitro	Adult and juvenile worms	5–80 μM	6 days	In adults, detachment of the sucker, couple dissociation, decrease in mobility, and elongation of body length.

#### 4.1.2. Bee (*Apis mellifera*)

Venoms from other arthropods have been tested against *S. mansoni*. A work published in 2016 [46] explored not only the effect of venom derived from the honeybee *Apis mellifera* on *S. mansoni*-infected mice, but also the effect of propolis. Bee venom is rich in enzymes, peptides, biogenic amines, and Phospholipase A2 (Pla2), while propolis, derived from plant resins and collected by the honeybees, is rich in complex substances, such as polyphenols, terpenoids, steroids, amino acids, and others [64].

The work recorded a reduction in the total worm burden and in the number of eggs in hepatic tissue in bee venom or propolis-treated groups as compared with the control group [46]. These results were attributed to the presence of several biocomponents that are present in the venom, including Pla2, which also has antiplasmodial activity [65] and might exert a potential role in oxidizing lipoproteins. Propolis, on the other hand, is known for its ability to modulate the cellular immune response and has been associated with severe damage to the tegument of other trematodes, such as *Fasciola gigantica* [66].

#### 4.1.3. Ladybird (*Harmonia axyridis*)

More recently, other animal-derived compounds have been tested (Table 2), such as harmonine, an antimicrobial alkaloid from the harlequin ladybird *Harmonia axyridis* [62]. This aliphatic compound, present in the ladybird hemolymph, had already been shown to have antimalarial activity [67]. In Kellershohn, Thomas [62], the group of Haeberlein tested concentrations from 2.5 to 50 μM on *S. mansoni* adult worms for up to 72 h. It was observed that, at 5 μM, 50% of the couples were separated and 90% detached, whereas, at concentrations of 20 and 50 μM, all worms died in 48 h and 2 h, respectively, showing a time- and dose-dependent effect, with an EC50 established at 8.8 μM. Moreover, concentrations as low as 5 μM produced bubbles on the tegumental surface and gut dilatation in both males and females, confirmed by confocal laser scanning microscopy.

By using an enzyme inhibition assay, this study suggested the inhibition of acetylcholinesterase (AChE) as a possible mode of action, which is abundant on the tegument of *S. mansoni* worms. RT-qPCR assays suggested a dysregulation of stem-cell gene expression [62]. Together, this makes harmonine the first animal-derived alkaloid detected to have an antischistosomal capacity. Of concern for future work is the potential cytotoxicity of harmonine to human cells, which was found against HepG2 cells at 50 µM, while lower concentrations were not toxic.

#### 4.1.4. Assassin Bug (*Rhynocoris iracundus*)

A similar approach was used by the group of Haeberlein in Tonk, Vilcinskas [48] to study the effects of the venom from the European assassin bug *Rhynocoris iracundus*. Assassin bugs are predatory hemipterans that belong to a family (*Reduviidae*) well-known for its powerful venom, injected with the help of a proboscis to paralyze its prey or as a defense mechanism [68]. *S. mansoni* adult worms were exposed for 72 h to 10 to 50 μg/mL of the venom. Besides a significant reduction in schistosome motility, *R. iracundus* venom reduced the pairing capacity and egg production of *S. mansoni* adult worms in vitro [48]. All worms were killed by 50 µg/mL (Figure 2A, column 3). One possible mode of action was the elimination of proliferative cells in the parasite. This resembles both the paralytic and the cytolytic effects evoked by assassin bug venom in its invertebrate prey [69].

A proteo-transcriptomic approach had assessed the venom‘s molecular composition [70]. The venom proteome consisted of up to 105 different proteins, many of them belonging to proteolytic toxin families (such as S1 proteases and trypsin-like proteases), cytolytic toxins (redulysins), and protease inhibitors (cystatin-like proteins) [70]. While some of these components may be potent in ablating cell proliferation in schistosomes, worm motility may be rather affected as a result of muscular or nervous dysfunction. This may involve the action of other interesting venom constituents, Ptu1 family peptides, which are known to act as calcium channel blockers [71] and, thus, may target nerve cells not only in the bug’s invertebrate prey, but also in flatworms.

Importantly, hemolytic side-effects at concentrations active against *S. mansoni* could be excluded, which would otherwise limit any therapeutic application. This motivates future efforts to identify the antischistosomal components in *R. iracundus* venom and to reveal their mode of action. Progress is, however, hampered by the fact that the manual collection of sufficient amounts of venom from assassin bugs for purposes such as fractionation is extremely laborious. This is a true drawback also for other animal-derived products, particularly from small animals such as insects.

#### 4.1.5. Jewel Beetle (*Chalcophora mariana*)

Most of the findings so far are derived from testing individual products or a limited number of animal-derived products against schistosomes in in vitro or in vivo models. This is typically time-consuming, as it involves the laborious harvesting of compounds from the animals without a guarantee of success in finding antiparasitic activity. Therefore, as an alternative, an in silico screening approach of more than 1300 insect-derived molecules against a proven druggable target protein has been applied for the first time [63]. By docking against the thioredoxin glutathione reductase of *S. mansoni* (SmTGR) [72], this approach identified several insect-derived molecules predicted to interact with key residues in a well-druggable pocket of SmTGR. Most of the top predicted compounds had known roles in the defense system of the related insect. This covered an antimicrobial molecule and a defensive alkaloid from beetles, and a molecule from neuroactive wasp venom, amongst others [63]. The approach of using a library of insect-derived molecules could serve as a basis for future in silico screenings against additional target proteins not only of schistosomes, but also of other parasites.

The in vitro activity against juvenile and adult *S. mansoni* was tested and confirmed for one molecule, buprestin H (Figure 2A, column 4), which represents an acyl glucose derivative from the European jewel beetle *Chalcophora mariana* [63] (see Table 2). While schistosomula were killed with 5–20 µM, only a moderate reduction in adult parasite motility was found for buprestin H (at 40 µM). No unspecific cytotoxic activity against human HepG2 cells was found [63].

#### 4.1.6. Ticks

Insects, as the most biodiverse groups of organisms, produce a large number of different AMPs as part of their humoral immune response, which accumulate in the insect hemolymph after microbial infection [73]. The same holds true for other arthropods, including ticks [74]. AMPs moved in focus as candidates for the treatment of drug-resistant microorganisms, and a few are already FDA-approved. To our knowledge, insect-derived AMPs, or AMPs of arthropod origin, in general, have not been tested for their antischistosomal potential before. Thirteen AMPs from different arthropod species were screened in vitro against the adult stage of *S. mansoni* at concentrations of up to 50 µM (unpublished data from Haeberlein and colleagues). This included ten insect-derived AMPs previously tested against *Plasmodium falciparum*, including drosocin from *Drosophila melanogaster* and various cecropins from the greater wax moth *Galleria mellonella* [48]. Furthermore, three defensins from *Ixodes* ticks were tested, which are the best studied class of AMPs in ticks [75,76].

While it was found that the insect AMPs had no antischistosomal effect or only affected the egg production of worm pairs, the defensin scapularisin-6 (Sca-6) from the black-legged tick *Ixodes scapularis* reduced worm motility at 50 µM (Figure 2A, column 5). Thus, the previously described antibacterial and antifungal activity of Sca-6 is now extended to act against *Schistosoma* parasites (Figure 2A, column 5). Given that Sca-6 had a rather specific activity against only some and not all pathogens under investigation [76], this might give hope that nonspecific cytotoxic effects against human cells can be excluded in future studies.

### 4.2. Marine-Invertebrate-Derived Compounds

#### Sea Cucumbers

Since the marine environment represents 70% of the earth’s surface, it emerges as one of the most valuable sources of active compounds. A screening of marine extracts for in vitro schistosomicidal activity was performed by Melek and collaborators [61]. Extracts from 79 different marine organisms were tested after maceration and filtration. The substances were in vitro incubated with adult *S. mansoni* worms, obtained by the perfusion of infected hamsters, which were observed daily for four days, in three replicates for each assay. Among the screened extracts, those obtained from the soft corals *Favia favus*, *Lobophytum pauciflorum*, *Sarcophyton digitatum*, *Sarcophyton spongiosum*, and *Sarcophyton glaucum*, the sponges *Cinachyrella tarentina*, *Clathria reinwardti*, and *Xestospongia testudinaria*, the gastropod *Cymatium aquatile*, the sea star *Ophiarachnella septemspinosa*, and the sea cucumbers *Actinopyga echinites*, *Holothuria nobilis*, and *Holothuria polii* demonstrated schistosomicidal effects on the worms [61].

The strongest antischistosomal capacity among the screened extracts was found in extracts of the sea cucumbers *A. echinites* and *H. polii* [61]. A subsequent isolation determined the most active compounds as two triterpene glycosides from these extracts, the echinosides A and B. The LC50 defined for these compounds were 0.19 and 0.27 μg/mL, respectively [61]. Triterpene glycosides had been previously reported to possess antifungal, hemolytic, and immunomodulatory activities [77].

Another work of research explored specific echinoderm-derived compounds: Mona and collaborators [36] tested extracts from three species of sea cucumbers against *S. mansoni*. Extracts from other sea cucumbers were already known for their effects against malaria parasites [78]. The authors prepared extracts from the tegument of the sea cucumbers *H. polii* (HPE) and *Actinopyga mauritiana* (AME), while, for *Bohadschia vitiensis*, the cuvierian gland was used (CGE), all of which contain a substance called holothurin [36]. These tissues underwent ethanol extraction and the holothurin extracts were administered orally to infected mice, with a significant decrease in the worm burden (60% in males and 90% in females) and in the liver egg count for mice treated with HPE, but not with AME and CGE [36]. In vitro treatment with all three extracts showed destructive effects on the tegument. These results support the hypothesis that holothurin can be considered a promising antischistosomal agent.

### 4.3. Vertebrate-Derived Compounds

#### 4.3.1. Snake Venoms

Among all venomous animals, snakes have been, for centuries, a wellspring of fascination and curiosity, and the therapeutic potential of snake venoms has been shown in different human diseases [79]. They differ consistently in their composition and complexity from other animal venoms, such as arthropods and molluscs. One single snake venom can contain over 100 components that can account for 90–95% of its dry weight [24] and its therapeutic effects are mainly caused by its multiple range of toxic proteins and peptides. Snake venoms include L-amino-acid oxidases, nucleases, hyaluronidases, snake venom metalloproteinases (SVMP), Pla2, and many other components [24]. Among those, the snake phospholipases are a class of cysteine-rich proteins, usually toxic, that contain 119–134 amino acids [80] involved in the digestion of the prey, in which they cause alterations in the cell membrane permeability by cleaving glycerophospholipids, releasing lysophospholipids and free fatty acids [81].

The antiparasitic effects of crude snake venoms have been mainly reported against different species of parasitic protozoans or microbes [82,83]. To date, antischistosomal tests with crude snake venoms or purified compounds are largely lacking. In *S. mansoni*, the myotoxic Phospholipase A2 homologue MjTX-II, obtained from *Bothrops moojeni* venom, was explored in the work of Stábeli and collaborators [45], which was the first attempt to use snake venoms as a source of therapeutics against *S. mansoni*. Besides in vitro antimicrobial and antitumor effects, MjTX-II reduced the egg burden in a mouse model of *S. mansoni* when administered at doses of 50–100 µg [45]. In addition, the authors emphasize the promotion of worm couple dissociation, inhibition of egg deposition, and death of the parasite. The induction of apoptosis was discussed as the cytotoxic mode of action [45]. Because MjTX-II induced different signs of toxicity in treated animals, it will be important to identify venom compounds with a more specific activity to qualify for development as a form of human therapeutics.

The only published study evaluating the effects of crude venom extract against schistosomes made use of venom from the horned viper snake *Cerastes cerastes* (family *Viperidae*) [47]. This member of the family *Viperidae* is medically important as it causes severe envenomation; the therapeutic potential of its venom has been discussed, including for antitumor and antibacterial purposes [84,85]. The venom of *C. cerastes* had an LC50 of 21.6 µg/mL against *S. mansoni* adult worms in vitro, and a dose- and time-dependent increase in worm mortality was noticed [47]. Interestingly, male worms were more affected by the venom compared with females [47]. Ultrastructural alterations were noticed by electron microscopy, such as the destruction of the oral sucker and of the tegument, including the loss and degradation of tubercle spines, formation of protuberances, and shortening of spines in the tegument that surround the gynaecophoric canal [47].

In an initiative to test the antischistosomal potential of snake venoms more systematically, the group of Haerberlein screened venom from six different snake species belonging to two medically important venomous groups, *Viperidae* and *Elapidae* (unpublished data). Amongst others, venom from the saw-scaled viper *Echis carinatus* reduced worm motility at 20 µg/mL (Figure 2A, column 2). The proteomic profiling of *E. carinatus* venom revealed SVMPs, serine proteinases, and Pla2 as the most abundant components, next to L-amino acid oxidases and C-type lectins/lectin-like protein families [86]. The compounds relevant for antischistosomal activity remain to be determined.

#### 4.3.2. Frogs

AMPs are found in most living organisms, including arthropods as discussed above, but also vertebrates. Besides potent antimicrobial activity, AMPs can fulfill various biological functions, including immune regulation, wound healing, and antitumor activity [87]. Consequently, AMPs are the focus of researchers as a source of novel agents for medical applications. AMPs are short, composed of less than 50 amino acids, and present amphipathic behavior. They are a defensive weapon from the host against pathogens due to their effects on the plasma membrane or intracellular targets [88]. A cationic AMP called Dermaseptin 01 (DS 01), found in the skin secretion of Brazilian frogs of the genus *Phyllomedusa*, was tested in vitro against *S. mansoni* adult worms [33].

DS 01 is one of more than 80 AMPs produced by the *Phyllomedusa* genus. Previous studies had already shown the in vitro activity of dermaseptins against other parasites, such as *Trypanosoma cruzi* [89], *Leishmania amazonensis* [90], *Leishmania chagasi* [91], and *Plasmodium* [92]. In the work against *S. mansoni* [33], DS 01 was synthesized using an automated bench-top simultaneous multiple solid-phase peptide synthesizer, and then purified. Different concentrations of the peptide were applied to adult worm pairs and both a time and dose dependence of DS 01 activity was noticed [33]. After 24 h, incubation with 200 mg/mL led to a 100% mortality rate, while, for doses of 100 and 50 mg/mL, 48 h and 120 h were necessary, respectively. No differences were found between male and female parasite mortality rates [33].

**Table 2:** Summarized information on animal-derived compounds showing antischistosomal activity.

Compound or Peptide	Description	Organism	Molecular Formula
MjTX-II [45]	Myotoxic Phospholipase A2 homologue	Snake (*Bothrops moojeni*)	-
Dermaseptin 01 [33]	Antimicrobial peptide	Frog (*Phyllomedusa*)	C_152_H_257_N_43_O_44_S_2_ [93]
Echinosides A and B [61]	Triterpene glycosides	Sea cucumbers (*A. echinites* and *H. polii*)	C_54_H_88_NaO_26_S^+^/ C_41_H_65_NaO_16_S [94,95]
Holothurin [36]	Glycoside	Sea cucumbers (*H. polii*, *B. vitiensis* and *A. mauritiana*).	-
Harmonine [62]	Antimicrobial alkaloid	Ladybird (*Harmonia axyridis*)	C_18_H_38_N_2_ [96]
Buprestin H [63]	Acyl glucose derivative	Jewel beetle (*Chalcophora mariana*)	C_24_H_24_N_2_O_10_ [97]
Scapularisin-6	Defensin peptide	Black-legged tick (*Ixodes scapularis*)	-

## 5. Conclusions

The struggle to find praziquantel substitutes is an urgent concern [98] and finding schistosome chemotherapy alternatives is a challenge for the drug discovery field. Natural products have been, for centuries, a source of therapeutics, especially in traditional medicine. Nevertheless, they have been underutilized by pharmaceutical companies, regardless of their historical contributions. The downsides of this source of compounds have contributed to the decrease in their use in drug discovery. The disadvantages include access restriction, their complex composition, concerns about intellectual property rights, and their inherent slowness of working with natural products [99]. In addition, anthelmintic drug discovery itself imposes difficulties, due to the parasite complex life cycles and low likelihood of experimental models [100]. In fact, most drug development studies focus on one stage, usually adult worms, while other key parasitic stages are neglected.

Animal-derived compounds include other significant challenges. These substances usually represent a high complex mixture, which demands screening knowledge for its optimization. Most of these compounds are obtained in very small quantities [52] and can be hard to find because of the animal distribution. Another important factor is the interaction of these compounds with the host metabolism. For instance, many venoms can induce necrosis [101], cause hemorrhagic damage [102], and lead to other complications. This way, the understanding of animal-derived compounds’ activity both in vitro and in vivo is essential. Finding a selective molecule must be a priority in order to minimize the possible harm to the host, despite the difficulty in isolating specific toxins. Even with many successful examples, there is still a gap between animal poisons and toxins pharmacologically discovered and those approved [52].

On the other hand, the rapid technological progress in molecular biology and bioinformatics permits the improvement in screening techniques. Virtual in silico screenings with candidate insect animal-derived products [63], if employed against other candidate target proteins, may reveal even more potent molecules in the future. Virtual screening approaches also of other animal-derived products may be fruitful; however, the availability of a molecule database containing structure codes, as well as stereochemistry information, is a prerequisite. For insect molecules, such a database was lacking. As part of the work from Haeberlein and colleagues, a pipeline was created to establish a suitable library based on the Dictionary of Natural Products [63]. Filtering a priori for small-molecule-like compounds in such screenings increases the chance for hits that are more likely to succeed in preclinical studies.

The availability of schistosome genomes is a valuable tool for identifying possible targets, which is important in delimiting selective molecules with a high efficacy [103,104]. The progress in the biotechnology field might permit the wide exploration of animal-derived compounds in drug discovery, despite its difficulties. Schistosome research was, so far, just scratching at the surface of the wealth of animal-derived products. Hence, further studies are indispensable in order to fully understand the chemotherapeutic potential of these substances, and to determine the suitability of them as therapeutics agents.

## Data Availability

The original contributions presented in this study are included in the article. Further inquiries can be directed to the corresponding author.

## Abbreviations

AChE	Acetylcholinesterase
AME	*Actinopyga mauritiana*
AMPs	Antimicrobial peptides
C.m.	*Chalcophora mariana*
CGE	Cuvierian gland of *Bohadschia vitiensis*
CLSM	Confocal laser scanning microscopy
DS 01	Dermaseptin 01
E.c.	*Echis carinatus*
EC50	Half maximal effective concentration
HPE	*Holothuria polii*
I.s.	*Ixodes scapularis*
LC50	Lethal concentration 50
MjTX-II	Myotoxic phospholipase A2 homologue from *Bothrops moojeni* venom
NP	Natural product
Pla2	Phospholipase A2
PZQ	Praziquantel
R.i.	*Rhynocoris iracundus*
RT-qPCR	Quantitative reverse transcription polymerase chain reaction
Sca-6	Scapularisin-6
SmTGR	Thioredoxin glutathione reductase from *Schistosoma mansoni*
SVMP	Snake venom metalloproteinase
WHO	World Health Organization

## References

[1] (2023). WHO. https://www.who.int/news-room/fact-sheets/detail/schistosomiasis.

[2] van der Werf M.J., de Vlas S.J., Brooker S., Looman C.W., Nagelkerke N.J., Habbema J.D., Engels D. (2003). Quantification of clinical morbidity associated with schistosome infection in sub-Saharan Africa. Acta Trop..

[3] Colley D.G., Bustinduy A.L., Secor W.E., King C.H. (2014). Human schistosomiasis. Lancet.

[4] Ezeamama A.E., He C.L., Shen Y., Yin X.P., Binder S.C., Campbell C.H., Rathbun S., Whalen C.C., N’Goran E.K., Utzinger J. (2016). Gaining and sustaining schistosomiasis control: Study protocol and baseline data prior to different treatment strategies in five African countries. BMC Infect. Dis..

[5] Gichuki P.M., Kepha S., Mulewa D., Masaku J., Kwoba C., Mbugua G., Mazigo H.D., Mwandawiro C. (2019). Association between *Schistosoma mansoni* infection and access to improved water and sanitation facilities in Mwea, Kirinyaga County, Kenya. BMC Infect. Dis..

[6] Ross A.G., Bartley P.B., Sleigh A.C., Olds G.R., Li Y., Williams G.M., McManus D.P. (2002). Schistosomiasis. N. Engl. J. Med..

[7] King C.H., Dickman K., Tisch D.J. (2005). Reassessment of the cost of chronic helmintic infection: A meta-analysis of disability-related outcomes in endemic schistosomiasis. Lancet.

[8] Li X.X., Zhou X.N. (2013). Co-infection of tuberculosis and parasitic diseases in humans: A systematic review. Parasit. Vectors.

[9] Secor W.E. (2012). The effects of schistosomiasis on HIV/AIDS infection, progression and transmission. Curr. Opin. HIV AIDS.

[10] Kura K., Hardwick R.J., Truscott J.E., Toor J., Hollingsworth T.D., Anderson R.M. (2020). The impact of mass drug administration on *Schistosoma haematobium* infection: What is required to achieve morbidity control and elimination?. Parasit. Vectors.

[11] Ismail M., Metwally A., Farghaly A., Bruce J., Tao L.F., Bennett J.L. (1996). Characterization of isolates of *Schistosoma mansoni* from Egyptian villagers that tolerate high doses of praziquantel. Am. J. Trop. Med. Hyg..

[12] Stelma F.F., Talla I., Polman K., Niang M., Sturrock R.F., Deelder A.M., Gryseels B. (1993). Epidemiology of *Schistosoma mansoni* infection in a recently exposed community in northern Senegal. Am. J. Trop. Med. Hyg..

[13] Kittur N., King C.H., Campbell C.H., Kinung’hi S., Mwinzi P.N.M., Karanja D.M.S., N’Goran E.K., Phillips A.E., Gazzinelli-Guimaraes P.H., Olsen A. (2019). Persistent Hotspots in Schistosomiasis Consortium for Operational Research and Evaluation Studies for Gaining and Sustaining Control of Schistosomiasis after Four Years of Mass Drug Administration of Praziquantel. Am. J. Trop. Med. Hyg..

[14] Melman S.D., Steinauer M.L., Cunningham C., Kubatko L.S., Mwangi I.N., Wynn N.B., Mutuku M.W., Karanja D.M., Colley D.G., Black C.L. (2009). Reduced susceptibility to praziquantel among naturally occurring Kenyan isolates of *Schistosoma mansoni*. PLoS Negl. Trop. Dis..

[15] Sanchez M.C., Cupit P.M., Bu L., Cunningham C. (2019). Transcriptomic analysis of reduced sensitivity to praziquantel in *Schistosoma mansoni*. Mol. Biochem. Parasitol..

[16] Coeli R., Baba E.H., Araujo N., Coelho P.M., Oliveira G. (2013). Praziquantel treatment decreases *Schistosoma mansoni* genetic diversity in experimental infections. PLoS Negl. Trop. Dis..

[17] Couto F.F., Coelho P.M., Araujo N., Kusel J.R., Katz N., Jannotti-Passos L.K., Mattos A.C. (2011). *Schistosoma mansoni*: A method for inducing resistance to praziquantel using infected *Biomphalaria glabrata* snails. Mem. Do Inst. Oswaldo Cruz.

[18] Le Clec’h W., Chevalier F.D., Mattos A.C.A., Strickland A., Diaz R., McDew-White M., Rohr C.M., Kinung’hi S., Allan F., Webster B.L. (2021). Genetic analysis of praziquantel response in schistosome parasites implicates a transient receptor potential channel. Sci. Transl. Med..

[19] World Health Organization (2023). The Weekly Epidemiological Record, Proceedings of the 34th meeting of the International Task Force for Disease Eradication, Atlanta, GA, USA, 19–20 September 2022.

[20] Atanasov A.G., Zotchev S.B., Dirsch V.M., International Natural Product Sciences T., Supuran C.T. (2021). Natural products in drug discovery: Advances and opportunities. Nat. Rev. Drug Discov..

[21] de Moraes J. (2015). Natural products with antischistosomal activity. Future Med. Chem..

[22] Neves B.J., Andrade C.H., Cravo P.V. (2015). Natural products as leads in schistosome drug discovery. Molecules.

[23] Herzig V., Cristofori-Armstrong B., Israel M.R., Nixon S.A., Vetter I., King G.F. (2020). Animal toxins—Nature’s evolutionary-refined toolkit for basic research and drug discovery. Biochem. Pharmacol..

[24] Mohamed Abd El-Aziz T., Garcia Soares A., Stockand J.D. (2019). Snake Venoms in Drug Discovery: Valuable Therapeutic Tools for Life Saving. Toxins.

[25] Newman D.J., Cragg G.M. (2020). Natural Products as Sources of New Drugs over the Nearly Four Decades from 01/1981 to 09/2019. J. Nat. Prod..

[26] Sulik M., Antoszczak M., Huczynski A., Steverding D. (2023). Antiparasitic activity of ivermectin: Four decades of research into a “wonder drug”. Eur. J. Med. Chem..

[27] Perez del Villar L., Burguillo F.J., Lopez-Aban J., Muro A. (2012). Systematic review and meta-analysis of artemisinin based therapies for the treatment and prevention of schistosomiasis. PLoS ONE.

[28] Correa S.A.P., de Oliveira R.N., Mendes T.M.F., Dos Santos K.R., Boaventura S., Garcia V.L., Jeraldo V.L.S., Allegretti S.M. (2019). In vitro and in vivo evaluation of six artemisinin derivatives against *Schistosoma mansoni*. Parasitol. Res..

[29] Lam N.S., Long X., Su X.Z., Lu F. (2018). Artemisinin and its derivatives in treating helminthic infections beyond schistosomiasis. Pharmacol. Res..

[30] Teodoro A., Goncalves F.J.M., Oliveira H., Marques S. (2022). Venom of *Viperidae*: A Perspective of its Antibacterial and Antitumor Potential. Curr. Drug Targets.

[31] Zouari-Kessentini R., Srairi-Abid N., Bazaa A., El Ayeb M., Luis J., Marrakchi N. (2013). Antitumoral potential of Tunisian snake venoms secreted phospholipases A2. Biomed. Res. Int..

[32] Radek K., Gallo R. (2007). Antimicrobial peptides: Natural effectors of the innate immune system. Semin. Immunopathol..

[33] de Moraes J., Nascimento C., Miura L.M., Leite J.R., Nakano E., Kawano T. (2011). Evaluation of the in vitro activity of dermaseptin 01, a cationic antimicrobial peptide, against *Schistosoma mansoni*. Chem. Biodivers..

[34] Chen C., Chen L., Mao C., Jin L., Wu S., Zheng Y., Cui Z., Li Z., Zhang Y., Zhu S. (2024). Natural Extracts for Antibacterial Applications. Small.

[35] Albaridi N.A. (2019). Antibacterial Potency of Honey. Int. J. Microbiol..

[36] Mona M.H., Omran N.E., Mansoor M.A., El-Fakharany Z.M. (2012). Antischistosomal effect of holothurin extracted from some Egyptian sea cucumbers. Pharm. Biol..

[37] El-Asmar M.F., Swelam N., Abdel Aal T.M., Ghoneim K., Hodhod S.S. (1980). Factor(s) in the venom of scorpions toxic to *Schistosoma mansoni* (intestinal belharzia) cercariae. Toxicon.

[38] Tempone A.G., Andrade H.F., Spencer P.J., Lourenco C.O., Rogero J.R., Nascimento N. (2001). *Bothrops moojeni* venom kills *Leishmania spp*. with hydrogen peroxide generated by its L-amino acid oxidase. Biochem. Biophys. Res. Commun..

[39] Borges A., Silva S., Op den Camp H.J., Velasco E., Alvarez M., Alfonzo M.J., Jorquera A., De Sousa L., Delgado O. (2006). In vitro leishmanicidal activity of *Tityus discrepans* scorpion venom. Parasitol. Res..

[40] Goncalves A.R., Soares M.J., de Souza W., DaMatta R.A., Alves E.W. (2002). Ultrastructural alterations and growth inhibition of *Trypanosoma cruzi* and *Leishmania major* induced by *Bothrops jararaca* venom. Parasitol. Res..

[41] Fernandez-Gomez R., Zerrouk H., Sebti F., Loyens M., Benslimane A., Ouaissi M.A. (1994). Growth inhibition of *Trypanosoma cruzi* and *Leishmania donovani infantum* by different snake venoms: Preliminary identification of proteins from *Cerastes cerastes* venom which interact with the parasites. Toxicon.

[42] Sabia Junior E.F., Menezes L.F.S., de Araujo I.F.S., Schwartz E.F. (2019). Natural Occurrence in Venomous Arthropods of Antimicrobial Peptides Active against Protozoan Parasites. Toxins.

[43] de Moura A.A., Kayano A.M., Oliveira G.A., Setubal S.S., Ribeiro J.G., Barros N.B., Nicolete R., Moura L.A., Fuly A.L., Nomizo A. (2014). Purification and biochemical characterization of three myotoxins from *Bothrops mattogrossensis* snake venom with toxicity against *Leishmania* and tumor cells. Biomed. Res. Int..

[44] Farias L.H.S., Rodrigues A.P.D., Coelho E.C., Santos M.F., Sampaio S.C., Silva E.O. (2017). Crotoxin stimulates an M1 activation profile in murine macrophages during *Leishmania amazonensis* infection. Parasitology.

[45] Stábeli R.G., Amui S.F., Sant’Ana C.D., Pires M.G., Nomizo A., Monteiro M.C., Romao P.R.T., Guerra-Sa R., Vieira C.A., Giglio J.R. (2006). *Bothrops moojeni* myotoxin-II, a Lys49-phospholipase A2 homologue: An example of function versatility of snake venom proteins. Comp. Biochem. Physiol. C Toxicol. Pharmacol..

[46] Mohamed A.H., Hassab El-Nabi S.E., Bayomi A.E., Abdelaal A.A. (2016). Effect of bee venom or proplis on molecular and parasitological aspects of *Schistosoma mansoni* infected mice. J. Parasit. Dis..

[47] Hassan E.A., Abdel-Rahman M.A., Ibrahim M.M., Soliman M.F. (2016). In vitro antischistosomal activity of venom from the Egyptian snake *Cerastes cerastes*. Rev. Soc. Bras. Med. Trop..

[48] Tonk M., Vilcinskas A., Grevelding C.G., Haeberlein S. (2020). Anthelminthic Activity of Assassin Bug Venom against the Blood Fluke *Schistosoma mansoni*. Antibiotics.

[49] Hardy M.C., Cochrane J., Allavena R.E. (2014). Venomous and poisonous Australian animals of veterinary importance: A rich source of novel therapeutics. Biomed. Res. Int..

[50] Escoubas P., King G.F. (2009). Venomics as a drug discovery platform. Expert. Rev. Proteom..

[51] Hayashi M.A., Camargo A.C. (2005). The Bradykinin-potentiating peptides from venom gland and brain of *Bothrops jararaca* contain highly site specific inhibitors of the somatic angiotensin-converting enzyme. Toxicon.

[52] Bordon K.C.F., Cologna C.T., Fornari-Baldo E.C., Pinheiro-Junior E.L., Cerni F.A., Amorim F.G., Anjolette F.A.P., Cordeiro F.A., Wiezel G.A., Cardoso I.A. (2020). From Animal Poisons and Venoms to Medicines: Achievements, Challenges and Perspectives in Drug Discovery. Front. Pharmacol..

[53] Oliveira A.L., Viegas M.F., da Silva S.L., Soares A.M., Ramos M.J., Fernandes P.A. (2022). The chemistry of snake venom and its medicinal potential. Nat. Rev. Chem..

[54] Matsui T., Fujimura Y., Titani K. (2000). Snake venom proteases affecting hemostasis and thrombosis. Biochim. Biophys. Acta.

[55] Paes Leme A.F., Prezoto B.C., Yamashiro E.T., Bertholim L., Tashima A.K., Klitzke C.F., Camargo A.C., Serrano S.M. (2008). *Bothrops* protease A, a unique highly glycosylated serine proteinase, is a potent, specific fibrinogenolytic agent. J. Thromb. Haemost..

[56] Oliveira A.K., Paes Leme A.F., Asega A.F., Camargo A.C., Fox J.W., Serrano S.M. (2010). New insights into the structural elements involved in the skin haemorrhage induced by snake venom metalloproteinases. Thromb. Haemost..

[57] Lee H., Jung E.S., Kang C., Yoon W.D., Kim J.S., Kim E. (2011). Scyphozoan jellyfish venom metalloproteinases and their role in the cytotoxicity. Toxicon.

[58] Gutierrez J.M., Escalante T., Rucavado A., Herrera C., Fox J.W. (2016). A Comprehensive View of the Structural and Functional Alterations of Extracellular Matrix by Snake Venom Metalloproteinases (SVMPs): Novel Perspectives on the Pathophysiology of Envenoming. Toxins.

[59] Zlotkin E., Miranda F., Lissitzky S. (1972). Proteins in scorpion venoms toxic to mammals and insects. Toxicon.

[60] Zlotkin E., Miranda F., Lissitzky S. (1972). A factor toxic to crustacean in the venom of the scorpion *Androctonus australis hector*. Toxicon.

[61] Melek F.R., Tadros M.M., Yousif F., Selim M.A., Hassan M.H. (2012). Screening of marine extracts for schistosomicidal activity in vitro. Isolation of the triterpene glycosides echinosides A and B with potential activity from the Sea Cucumbers *Actinopyga echinites* and *Holothuria polii*. Pharm. Biol..

[62] Kellershohn J., Thomas L., Hahnel S.R., Grunweller A., Hartmann R.K., Hardt M., Vilcinskas A., Grevelding C.G., Haeberlein S. (2019). Insects in anthelminthics research: Lady beetle-derived harmonine affects survival, reproduction and stem cell proliferation of *Schistosoma mansoni*. PLoS Negl. Trop. Dis..

[63] Gallinger T.L., Aboagye S.Y., Obermann W., Weiss M., Grunweller A., Unverzagt C., Williams D.L., Schlitzer M., Haeberlein S. (2022). First In Silico Screening of Insect Molecules for Identification of Novel Anti-Parasitic Compounds. Pharmaceuticals.

[64] Kartal M., Yildiz S., Kaya S., Kurucu S., Topcu G. (2003). Antimicrobial activity of propolis samples from two different regions of Anatolia. J. Ethnopharmacol..

[65] Guillaume C., Calzada C., Lagarde M., Schrevel J., Deregnaucourt C. (2006). Interplay between lipoproteins and bee venom phospholipase A2 in relation to their anti-*Plasmodium* toxicity. J. Lipid Res..

[66] Hegazi A.G., Abd El Hady F.K., Shalaby H.A. (2007). An in vitro effect of propolis on adult worms of *Fasciola gigantica*. Vet. Parasitol..

[67] Rohrich C.R., Ngwa C.J., Wiesner J., Schmidtberg H., Degenkolb T., Kollewe C., Fischer R., Pradel G., Vilcinskas A. (2012). Harmonine, a defence compound from the harlequin ladybird, inhibits mycobacterial growth and demonstrates multi-stage antimalarial activity. Biol. Lett..

[68] Walker A.A., Weirauch C., Fry B.G., King G.F. (2016). Venoms of Heteropteran Insects: A Treasure Trove of Diverse Pharmacological Toolkits. Toxins.

[69] Walker A.A., Madio B., Jin J., Undheim E.A., Fry B.G., King G.F. (2017). Melt With This Kiss: Paralyzing and Liquefying Venom of The Assassin Bug *Pristhesancus plagipennis* (Hemiptera: *Reduviidae*). Mol. Cell Proteom..

[70] Rugen N., Jenkins T.P., Wielsch N., Vogel H., Hempel B.F., Sussmuth R.D., Ainsworth S., Cabezas-Cruz A., Vilcinskas A., Tonk M. (2021). Hexapod Assassins’ Potion: Venom Composition and Bioactivity from the Eurasian Assassin Bug *Rhynocoris iracundus*. Biomedicines.

[71] Bernard C., Corzo G., Mosbah A., Nakajima T., Darbon H. (2001). Solution structure of Ptu1, a toxin from the assassin bug *Peirates turpis* that blocks the voltage-sensitive calcium channel N-type. Biochemistry.

[72] Kuntz A.N., Davioud-Charvet E., Sayed A.A., Califf L.L., Dessolin J., Arner E.S., Williams D.L. (2007). Thioredoxin glutathione reductase from *Schistosoma mansoni*: An essential parasite enzyme and a key drug target. PLoS Med..

[73] Manniello M.D., Moretta A., Salvia R., Scieuzo C., Lucchetti D., Vogel H., Sgambato A., Falabella P. (2021). Insect antimicrobial peptides: Potential weapons to counteract the antibiotic resistance. Cell Mol. Life Sci..

[74] Hajdusek O., Sima R., Ayllon N., Jalovecka M., Perner J., de la Fuente J., Kopacek P. (2013). Interaction of the tick immune system with transmitted pathogens. Front. Cell Infect. Microbiol..

[75] Tonk M., Cabezas-Cruz A., Valdes J.J., Rego R.O., Grubhoffer L., Estrada-Pena A., Vilcinskas A., Kotsyfakis M., Rahnamaeian M. (2015). *Ixodes ricinus* defensins attack distantly-related pathogens. Dev. Comp. Immunol..

[76] Tonk M., Cabezas-Cruz A., Valdes J.J., Rego R.O., Chrudimska T., Strnad M., Sima R., Bell-Sakyi L., Franta Z., Vilcinskas A. (2014). Defensins from the tick *Ixodes scapularis* are effective against phytopathogenic fungi and the human bacterial pathogen *Listeria grayi*. Parasit. Vectors.

[77] Chludil H.D., Muniain C.C., Seldes A.M., Maier M.S. (2002). Cytotoxic and antifungal triterpene glycosides from the Patagonian sea cucumber *Hemoiedema spectabilis*. J. Nat. Prod..

[78] Yoshida S., Shimada Y., Kondoh D., Kouzuma Y., Ghosh A.K., Jacobs-Lorena M., Sinden R.E. (2007). Hemolytic C-type lectin CEL-III from sea cucumber expressed in transgenic mosquitoes impairs malaria parasite development. PLoS Pathog..

[79] Waheed H., Moin S.F., Choudhary M.I. (2017). Snake Venom: From Deadly Toxins to Life-saving Therapeutics. Curr. Med. Chem..

[80] Samy R.P., Gopalakrishnakone P., Stiles B.G., Girish K.S., Swamy S.N., Hemshekhar M., Tan K.S., Rowan E.G., Sethi G., Chow V.T. (2012). Snake venom phospholipases A(2): A novel tool against bacterial diseases. Curr. Med. Chem..

[81] Cedro R.C.A., Menaldo D.L., Costa T.R., Zoccal K.F., Sartim M.A., Santos-Filho N.A., Faccioli L.H., Sampaio S.V. (2018). Cytotoxic and inflammatory potential of a phospholipase A(2) from *Bothrops jararaca* snake venom. J. Venom. Anim. Toxins Incl. Trop. Dis..

[82] Perumal Samy R., Stiles B.G., Franco O.L., Sethi G., Lim L.H.K. (2017). Animal venoms as antimicrobial agents. Biochem. Pharmacol..

[83] Salimo Z.M., Barros A.L., Adriao A.A.X., Rodrigues A.M., Sartim M.A., de Oliveira I.S., Pucca M.B., Baia-da-Silva D.C., Monteiro W.M., de Melo G.C. (2023). Toxins from Animal Venoms as a Potential Source of Antimalarials: A Comprehensive Review. Toxins.

[84] Zouari-Kessentini R., Luis J., Karray A., Kallech-Ziri O., Srairi-Abid N., Bazaa A., Loret E., Bezzine S., El Ayeb M., Marrakchi N. (2009). Two purified and characterized phospholipases A2 from *Cerastes cerastes* venom, that inhibit cancerous cell adhesion and migration. Toxicon.

[85] Hanane-Fadila Z.M., Fatima L.D. (2014). Purification, characterization and antibacterial activity of L-amino acid oxidase from *Cerastes cerastes*. J. Biochem. Mol. Toxicol..

[86] Ghezellou P., Albuquerque W., Garikapati V., Casewell N.R., Kazemi S.M., Ghassempour A., Spengler B. (2021). Integrating Top-Down and Bottom-Up Mass Spectrometric Strategies for Proteomic Profiling of Iranian Saw-Scaled Viper, *Echis carinatus sochureki*, Venom. J. Proteome Res..

[87] Hassan M., Flanagan T.W., Kharouf N., Bertsch C., Mancino D., Haikel Y. (2023). Antimicrobial Proteins: Structure, Molecular Action, and Therapeutic Potential. Pharmaceutics.

[88] Ladram A., Nicolas P. (2016). Antimicrobial peptides from frog skin: Biodiversity and therapeutic promises. Front. Biosci..

[89] Brand G.D., Leite J.R., Silva L.P., Albuquerque S., Prates M.V., Azevedo R.B., Carregaro V., Silva J.S., Sa V.C., Brandao R.A. (2002). Dermaseptins from *Phyllomedusa oreades* and *Phyllomedusa distincta*. Anti-*Trypanosoma cruzi* activity without cytotoxicity to mammalian cells. J. Biol. Chem..

[90] Brand G.D., Leite J.R., de Sa Mandel S.M., Mesquita D.A., Silva L.P., Prates M.V., Barbosa E.A., Vinecky F., Martins G.R., Galasso J.H. (2006). Novel dermaseptins from *Phyllomedusa hypochondrialis* (Amphibia). Biochem. Biophys. Res. Commun..

[91] Zampa M.F., Araujo I.M., Costa V., Nery Costa C.H., Santos J.R., Zucolotto V., Eiras C., Leite J.R. (2009). Leishmanicidal activity and immobilization of dermaseptin 01 antimicrobial peptides in ultrathin films for nanomedicine applications. Nanomedicine.

[92] Dagan A., Efron L., Gaidukov L., Mor A., Ginsburg H. (2002). In vitro antiplasmodium effects of dermaseptin S4 derivatives. Antimicrob. Agents Chemother..

[93] National Center for Biotechnology Information PubChem Compound Summary for CID 16130489, Dermaseptin 2024. https://pubchem.ncbi.nlm.nih.gov/compound/Dermaseptin.

[94] National Center for Biotechnology Information PubChem Compound Summary for CID 156831, Echinoside A 2024. https://pubchem.ncbi.nlm.nih.gov/compound/Echinoside-A.

[95] National Center for Biotechnology Information PubChem Compound Summary for CID 73999936, CID 73999936, Echinoside B 2024. https://pubchem.ncbi.nlm.nih.gov/compound/73999936.

[96] Chemspider. CSID:29353985, Harmonine 2024. https://www.chemspider.com/Chemical-Structure.29353985.html.

[97] Ryczek S., Dettner K., Unverzagt C. (2009). Synthesis of buprestins D, E, F, G and H; structural confirmation and biological testing of acyl glucoses from jewel beetles (Coleoptera: Buprestidae). Bioorg Med. Chem..

[98] Vale N., Gouveia M.J., Rinaldi G., Brindley P.J., Gärtner F., Correia da Costa J.M. (2017). Praziquantel for Schistosomiasis: Single-Drug Metabolism Revisited, Mode of Action, and Resistance. Antimicrob. Agents Chemother..

[99] Harvey A.L. (2008). Natural products in drug discovery. Drug Discov. Today.

[100] Mackenzie C.D., Geary T.G. (2013). Addressing the current challenges to finding new anthelminthic drugs. Expert. Rev. Anti Infect. Ther..

[101] Harris J.B., Cullen M.J. (1990). Muscle necrosis caused by snake venoms and toxins. Electron. Microsc. Rev..

[102] Escalante T., Rucavado A., Fox J.W., Gutierrez J.M. (2011). Key events in microvascular damage induced by snake venom hemorrhagic metalloproteinases. J. Proteom..

[103] Berriman M., Haas B.J., LoVerde P.T., Wilson R.A., Dillon G.P., Cerqueira G.C., Mashiyama S.T., Al-Lazikani B., Andrade L.F., Ashton P.D. (2009). The genome of the blood fluke *Schistosoma mansoni*. Nat..

[104] Luo F., Yang W., Yin M., Mo X., Pang Y., Sun C., Zhu B., Zhang W., Yi C., Li Z. (2022). A chromosome-level genome of the human blood fluke *Schistosoma japonicum* identifies the genomic basis of host-switching. Cell Rep..

